# Inhibiting the Physiological Stress Effects of a Sustained Attention Task on Shoulder Muscle Activity

**DOI:** 10.3390/ijerph15010115

**Published:** 2018-01-11

**Authors:** Fiona Wixted, Cliona O’Riordan, Leonard O’Sullivan

**Affiliations:** 1 School of Design & Health Research Institute, University of Limerick, V94 T9PX Limerick, Ireland; Leonard.osullivan@ul.ie; 2 Department of Clinical Therapies, University of Limerick, V94 T9PX Limerick, Ireland; cliona.oriordan@ul.ie

**Keywords:** vigilance, musculoskeletal system, work physiology, industrial/workplace ergonomics, job stress

## Abstract

Objective: The objective of this study was to investigate if a breathing technique could counteract the effects of hyperventilation due to a sustained attention task on shoulder muscle activity. Background: The trend towards higher levels of automation in industry is increasing. Consequently, manufacturing operators often monitor automated process for long periods of their work shift. Prolonged monitoring work requires sustained attention, which is a cognitive process that humans are typically poor at and find stressful. As sustained attention becomes an increasing requirement of manufacturing operators’ job content, the resulting stress experienced could contribute to the onset of many health problems, including work related musculoskeletal disorders (WRMSDs). Methods: The SART attention test was completed by a group of participants before and after a breathing intervention exercise. The effects of the abdominal breathing intervention on breathing rate, upper trapezius muscle activity and end-tidal CO_2_ were evaluated. Results: The breathing intervention reduced the moderation effect of end-tidal CO_2_ on upper trapezius muscle activity. Conclusions: Abdominal breathing could be a useful technique in reducing the effects of sustained attention work on muscular activity. Application: This research can be applied to highly-automated manufacturing industries, where prolonged monitoring of work is widespread and could, in its role as a stressor, be a potential contributor to WRMSDs.

## 1. Introduction

Automation continues to be a global trend with an estimate that about 1.3 million new industrial robots will be installed in factories around the world between 2015 and 2018 [1]. This growth in automation has led to increased mental workload requirements for operators, in the form of prolonged vigilance work [2] requiring high levels of sustained attention. Many studies have indicated that sustained attention tasks induce stress [3,4,5]. Attentional resource or overload theories view vigilance work as being a source of extensive mental workload which quickly depletes cognitive resources [6]. Conflicting theories also exist which consider attention work to be under-stimulating and not highly demanding on cognitive resources. Some of these, including the mind-wandering theory and the mindlessness theory, suggest that a mindless approach is taken to monotonous monitoring work that leads to less focus on the task and more on internal thoughts [7,8]. While both sets of theories have validity in different respects, the growing body of empirical research supporting resource theories gives them increasing credibility. The current study is based on overload theories which view vigilance work as stressful. Many experimental studies have supported overload theories, finding that sustained attention tasks result in physiological stress responses including skin temperature [4], electrodermal activity [9], heart rate variability [10], blink rate [11] and electroencephalography (EEG) [3,11]. The adverse health effects of prolonged monitoring work are likely to stem from a chronic physiological stress response which can contribute to various health problems, e.g., cardiovascular and musculoskeletal conditions.

Work-related stress was found to be the second-most common occupational health problem across the EU15 [12]. Psychosocial factors which have the potential for causing psychological or physical harm include job content, workload, pace, work schedule, environment and equipment, job control, and job demands [13]. As sustained attention is a job demand and a potential source of stress, it presents a psychosocial risk. Psychosocial risks contribute to numerous occupational health problems, one being Work-Related Musculoskeletal Disorders (WRMSDs) [14,15]. To date, no study to our knowledge has looked at the potential contribution of sustained attention work to musculoskeletal disorders.

The term musculoskeletal disorder denotes health problems of the musculoskeletal system, i.e., muscles, tendons, the skeleton, cartilage, the vascular system, ligaments, and nerves [16]. WRMSDs are currently the most common occupational health problem in Europe [17]. The Fifth European Working Conditions Survey found that musculoskeletal disorders directly related to physical working conditions are in decline, while WRMSDs related to stressful working environments are increasing [18]. Psychosocial stressors can contribute to the aetiology of WRMSDs, but can also solely trigger their development by contributing to increases in muscular activity [19]. Numerous studies support links between psychosocial risks and increases in muscular activity [20,21]. The hyperventilation theory links work-related stress to WRMSDs via a dysfunctional breathing pattern known as hyperventilation [22]. This theory proposes that hyperventilation is a mediator or necessary factor to be present for stress to impact muscular health. A mediating variable is one that explains the relationship between the independent variable (IV) and the dependent variable (DV), with stress being the IV and muscular activity being the DV in the context of the hyperventilation theory.

It is widely accepted that stress can lead to hyperventilation or breathing more air than required for metabolism [23]. Taking quicker breaths as occurs in hyperventilation exchanges more of the alveolar gas with ambient air than in normal breathing. Thereby more CO_2_ is expelled from the body and the remaining lowered concentration of CO_2_ leads to blood respiratory alkalosis (pH > 7.45), which is known as hypocapnia [24]. Carbon dioxide has an essential function in maintaining the bodys’ pH balance, and even minor imbalances can influence the immune, endocrine system, sensations of pain, and muscle function. Hyperventilation can be measured by detecting a decrease in the concentration of end-tidal CO_2_ (PetCO_2_) which is the concentration of CO_2_ at the end of an exhaled breath [25]. If the end-tidal CO_2_ is less than 35 mmHg [26] the subject is in a state of hypocapnia, which may be due to hyperventilation. The resulting disruption in acid-base equilibrium triggers neuronal excitation, causing increased muscle tension, and spasms with adverse effects for muscle tissue [27]. Schliefer et al. [22] posited that hyperventilation was a mediator in the relationship between job stress and musculoskeletal disorders. Recent research by the current authors suggests that hyperventilation is, in fact, a moderator (variable that effects the direction/strength of the relation between two variables) of this relationship, where hyperventilation strengthens the effect of stress on muscle activity [28]. If hyperventilation is a moderator, it can be prevented/reduced by improving ones respiration pattern. This, in turn, would help to reduce sustained muscle activity, a risk factor in the development of musculoskeletal disorders.

Respiration is sensitive to cognitive workload. The literature to date has shown evidence for a relationship between mental stress and end-tidal CO_2_. Greater reductions in end-tidal CO_2_ were found in students who were anxious above those that weren’t during a memory test [29]. End-tidal CO_2_ was found to successfully differentiate between three cognitive test conditions when respiration rate and interbeat interval could not [30]. A further study by Schleifer et al. [31] proposed that lowered end-tidal CO_2_ may mediate trapezius muscle activity during computer data entry tasks.

Hyperventilation also imposes a biomechanical load on the neck/shoulder region as a direct result of the shift from abdominal to chest breathing [32,33]. Chest breathing is the most common type of dysfunctional breathing and upper trapezius muscles along with pectoralis major, pectoralis minor, and latissimus dorsi muscles, though not needed for respiration, become active during chest breathing [34].

Behavioural interventions that include paced breathing (slow and deep breathing) have been found to elicit improvements in many health conditions. These include stress [35], anxiety [36], asthma [37], chronic hyperventilation [38], and congestive heart failure [39]. The autonomic nervous system, which regulates the key involuntary functions of the body comprises two parts: the sympathetic nervous system and the parasympathetic nervous system. The sympathetic nervous system speeds up heart rate and other physiological functions in response to a stressor, while the parasympathetic nervous system is responsible for controlling homeostasis and restoring the body to a state of calm allowing it to relax and repair. There is evidence to suggest that slow breathing has positive effects on the autonomic nervous system in both the short [40,41] and long-term [42]. For example, studies using short breathing exercises have produced acute physiologic improvements (e.g., reduced cardiovascular and muscular activation) and reductions in symptoms of stress and anxiety [43,44]. A long-term study which focused on slowed breathing resulted in reduced cardiovascular activation and risk of cardiovascular disease [42].

The overall aim of slow breathing exercises is to reduce respiratory frequency and depth of breathing to match alveolar ventilation to metabolic demand [43]. Respiration rate is the number of breathing cycles in a minute, and healthy people breathe at a rate of 10–20 breaths per minute. Reduction of the breathing rate from 16 breaths/min to less than 10 breaths/min for at least two minutes was found to enhance parasympathetic system dominance aiding the body to stay relaxed and reduce sympathetic activation in a study group [44]. Although these changes fade on return to a normal breathing rate, a decreased tendency to increase respiration rate when exposed to stress was observed in slow breathing trainees who learned to breathe slowly and deeply [45].

While links have been previously established between many psychosocial stressors and WRMSDs, to our knowledge, no study to date has looked at sustained attention as a psychosocial stressor which may contribute to increased muscular activity, and ultimately the development of WRMSDs. The hyperventilation theory of job stress and musculoskeletal disorders [22] posits that hyperventilation plays a mediating role in the relationship between stress and muscle activity, but recent experimental work shows that it, is in fact, likely to be a moderator of this relationship [28]. The focus of this observational study was on investigating if a breathing intervention could reduce the muscular activity during sustained attention work by weakening the moderating effect of hyperventilation. The study aimed to establish an association between slowed breathing and decreased muscular activity while carrying out a sustained attention task.

## 2. Methods

### 2.1. Participants

Twenty-four healthy student participants from a variety of disciplines, 13 female and 11 male, with an average age of 18.6 years took part in the study. Participation was incentivised with a €30 voucher. Individuals with a history of musculoskeletal or respiratory illness and smokers were excluded from the study. Participants were asked to refrain from caffeine for at least 2 h prior to taking part in the study. This research complied with the American Psychological Association Code of Ethics and was approved by the University of Limerick Research Ethics Committee. Informed consent was obtained from each participant.

### 2.2. Study Design

An observational study design was used, whereby the dependent variables were measured under three conditions: (1) baseline (normal breathing); (2) while performing a sustained attention task (Sustained Attention to Response Task, SART1); and (3) performing the sustained attention task, after performing a breathing protocol (Sustained Attention to Response Task SART2).

#### 2.2.1. Dependent Variable

The primary dependent variable was upper trapezius muscle activity (UTMA). UTMA was assessed because increases in shoulder muscle activity have been linked to mental demands [46], attention [47], and emotional stress [48].

#### 2.2.2. Moderator Variable

PetCO_2_ was measured as a moderator variable. A variable functions as a moderator to the extent that it affects the relation (direction and/or strength) between an independent and a dependent variable. Essentially, a moderator modifies the strength or direction (i.e., positive or negative) of a causal relationship. A suitable analogy for a moderator is a dimmer that adjusts the strength at which one can switch on the lighting.

In the context of this study, PetCO_2_ concentrations <35 mmHg—where hyperventilation occurs [26] represent the conditions under which the effect of inhibited parasympathetic activity on upper trapezius muscle activity becomes maximally effective. In other words, it shows us that hyperventilation in its role as a moderator can strengthen the relationship between attention-related stress and UTMA.

#### 2.2.3. Independent Variable

The independent variable was the stress experienced while carrying out an attention task. Stress resulting from the attention task was measured (post-task) using the ‘distress’ and ‘worry’ subscales of the Short State Stress Questionnaire (SSSQ) [49]. This is a 24-item scale based on the longer 96-item Dundee State Stress Questionnaire (DSSQ) [50].

The SSSQ has been employed in several industrial studies due to its shorter length [51,52,53]. In the first part of this questionnaire respondents are asked to rate (using a Likert scale from 1 to 5) how they feel at that moment using 10 listed emotions. In Section 2 of the questionnaire, respondents are asked to rate using a Likert scale between 1 and 5, the extent to which 14 listed statements relating to their work apply to them, e.g., ‘I am motivated to do the task’. The negative stress states ‘distress’ and ‘worry’ were used in this study.

##### Sustained Attention to Response Test

The attention task performed was the Sustained Attention to Response Test (SART) [54]. The SART is a simple reaction time task constructed so that it leads to an automatic tendency to respond to every stimulus. On a few unexpected occasions participants have to inhibit their response. It was chosen as a prolonged, simple, monotonous, repetitive task, which has similar characteristics to tasks being monitored in industrial settings. Although this task was developed as a sustained attention task by a proponent of the mindlessness theory of vigilance, it has been found to be a cognitively demanding task in several studies [27,55,56]. Some theorists have found this test to be a good measure of response inhibition [57,58], but it also remains to be a widely-used sustained-attention test [59,60]. It involved 675 single digits (75 of each of the nine digits) being presented visually for a 12.9 min duration. Each digit was presented for 250 ms, followed by a 900 ms mask. Participants responded with a ‘Y’ key press of their dominant hand to each digit, except for 75 occasions on which the digit 3 appeared, when they had to withhold a response. The digits were presented in one of five randomly allocated font sizes to enhance the demands for processing the numerical value (48, 72, 94, 100, and 120 point, respectively). The mask following each digit consisted of a circle with a diagonal cross in the middle (diameter 29 mm). Both digits and mask were presented centrally on white, against a black computer screen. The contrast ratio between the lines of the mask and the black background was 92% (Michaelson’s Equation for Spatial Modulation). The SART ran for 12.9 min. The SART task performed after baseline is termed SART1 and after the breathing protocol is termed SART2.

### 2.3. Experimental Measurements

#### 2.3.1. Upper Trapezius Muscle Activity

UTMA was measured using surface electromyography (EMG) electrodes on the non-dominant upper trapezius muscle, centred on a point 2 cm lateral to the midpoint between the acromion process, and the spinous process of the seventh cervical vertebra [47]. A bipolar configuration was employed with a centre to centre inter-electrode distance of 20 mm. Active electrodes were referenced to a ground electrode placed over the radius bone on the subjects’ non-dominant inner wrist. Surface electromyography was recorded using disposable, pre-gelled dual Ag/AgCl electrodes (Covidien H124SG 24 mm). EMG signals were recorded using a Nexus 10 (Mind Media BV, Herten, The Netherlands) physiological monitoring and feedback system set at a sampling rate of 2048 Hz. For data analysis, raw root mean square (RMS) values were averaged over 1/16 s epochs using the Biotrace software programme. The band pass DC corrections were set at 0.5 Hz. A digital fourth-order Butterworth cascaded IIR band pass filter was set to 20–500 Hz.

#### 2.3.2. Hyperventilation (PetCO_2_)

PetCO_2_ (mmHg) was measured with a face piece connected to a capnography device (CosMED K4B^2^ physiological measurement system) [23]. This system is a sidestream capnograph where the exhaled CO_2_ is aspirated via a mask through a sampling tube connected to the instrument for analysis. Samples were recorded every 1 s and averaged over each task. The partial pressure of PetCO_2_ is highly correlated with alveolar pCO_2_, which is regarded as a valid estimate of arterial pCO_2_. The normal values are 5% to 6% CO_2_, which is equivalent to 35–45 mmHg [61].

#### 2.3.3. Breathing Rate

A Nexus respiration sensor was placed around the abdomen of each participant to monitor abdominal breathing activity. This sensor is incorporated into a belt, which is placed around the abdomen, with the central part of the sensor placed just above the navel. The sensor measures the relative expansion and contraction of the abdomen. It was also logged with the NEXUS 10 physiological monitoring system at a sampling rate of 32 Hz.

### 2.4. Procedure

#### 2.4.1. Study Protocol

The procedure involved participants sitting at a computer workstation with their dominant arm placed with the elbow flexed 90°, their forearm resting on the workstation surface, and their hand positioned on the computer keyboard. The screen was positioned approximately 40 cm from their eyes.

The test protocol involved four stages, which participants performed in the same order. The first one was a baseline measurement condition where the dependent variables were measured while participants were idle (12 min). Participants sat in front of a blank computer screen and breathed normally, in a neutral posture. The second stage required the participants to perform SART1 on the computer (12.9 min). The third stage involved the breathing intervention (30 min), followed by the fourth stage where the participants repeated the SART test (SART2) (12.9 min). The SSSQ questionnaire was completed by each participant after SART1 and again after SART2.

#### 2.4.2. Breathing Protocol

The breathing protocol was used to counter inhibition of the parasympathetic system after the acute stress of the attention test, with the prediction of restricting an increase in muscle activity. This involved teaching participants a simple abdominal breathing technique at the start of the breathing intervention [62,63]. They were instructed to drop their shoulders, close their eyes, and release any tension from the body. They were then requested to place one hand on the abdomen and one the chest, and to inhale deeply and slowly through the nose into the abdomen. This was followed by exhaling through the mouth keeping the mouth, tongue, and jaw relaxed. Participants were asked to open their eyes and take deep and long controlled breaths while making the breath smooth and even. They performed the abdominal breathing technique for a 30 min period while viewing a relaxing image on a PC screen [33].

### 2.5. Study Hypotheses

Hypothesis: A short abdominal breathing protocol can reduce the moderation effect of hyperventilation (PetCO_2_) on the relationship between stress induced by sustained attention, and upper trapezius muscle activity.

### 2.6. Statistical Analysis

To test the hypothesis, basic moderation models based on the results of SART1 and SART2 tasks were investigated. Moderation is assessed by way of interaction effects in regression models. In this study, model 1 (based on the SART1 test results) and model 2 (based on the SART2 results) were used to determine when PetCO_2_ moderates the relationship between stress and upper trapezius muscle activity. This analysis was performed using an SPSS macro ‘PROCESS’, developed by Hayes [64]. This macro generates a multiple linear regression-based path analysis resulting in a moderation model.

**Figure 1:**
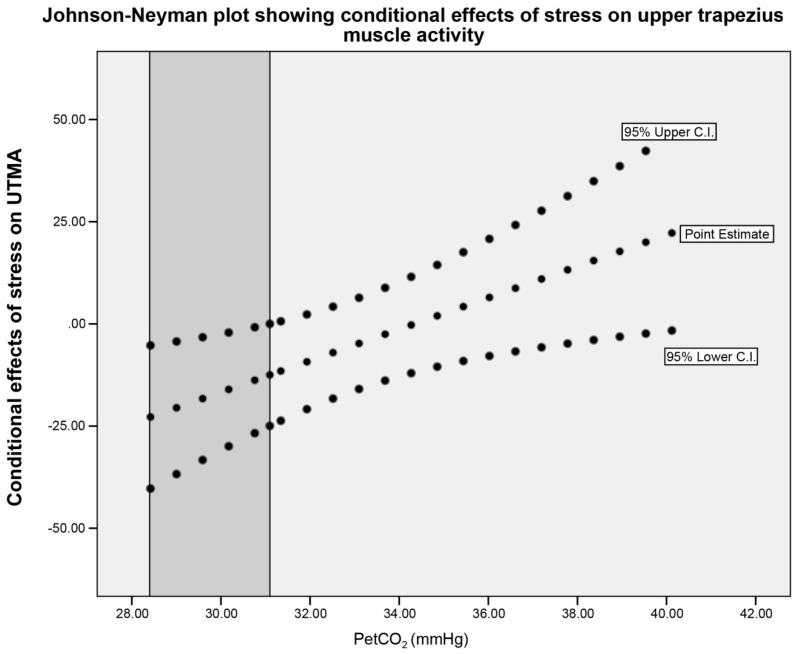
Johnson-Neyman graph showing the range of PetCO_2_ values where significant effects of stress on upper trapezius muscle activity occurred.

The Johnson-Neyman technique [65] (Figure 1) was used to probe the interaction between stress and PetCO_2_ for the SART1 task. This technique identifies the values within the measurement range of the moderator where the conditional effect of the independent variable (stress) transitions between statistically significant and not significant. Significance was set at the 5% level. Statistical analysis was performed using the SPSS 22.0 statistical package (IBM Corporation, Armonk, NY, USA) along with the ‘PROCESS’ macro for moderation analysis. The distress and worry subscales of the SSSQ questionnaire were analysed using factor analysis with the SPSS 22.0 statistical package. The distress and worry scores were then combined for each participant to give an overall score for sustained attention-related stress. The required sample size was predicted (N = 23) using the G-power statistical software package using an effect size of 0.6 (F-test, ᾳ = 0.05, predictors = 3) based on previous experimental research [28].

## 3. Results

Table 1 details the descriptive statistics for the dependent variables across the three study conditions (Baseline, SART1, and SART2). UTMA and breathing rate increased from the baseline during SART1 and decreased during SART2 after the breathing protocol as expected. This decrease in PetCO_2_ from baseline to SART1, and again during the SART2 test was not anticipated. Table 1 also shows the differences between the three test conditions, Baseline, SART1, and SART2 for the main study variables. As subjective stress scores were not collected for the baseline condition, a *t*-test was used to analyse differences between SART1 and SART2 for this variable. The results show a significant difference for UTMA, breathing rate and PetCO_2_ across the conditions. There was no significant difference for stress. Although breathing rate was not included in the statistical model, it was monitored to ensure the breathing intervention was effective and as an additional variable to show the relaxing effect of the slowed breathing.

Table 2 shows the mean and standard deviation for each variable during the SART2 test. This table splits the participants into Group A (N = 12) and Group B (N = 12). An increase in PetCO_2_ was evident in SART2 compared to the SART1 results in Group A, which suggests that the breathing intervention had a relaxing effect for this group. Group B participants showed a decrease in PetCO_2_ levels from SART1 to SART2 indicating the breathing intervention did not have the effect for this group.

Table 3 details the results of the moderation models for SART1 and SART2. A significant moderation model analysis indicates that the level of PetCO_2_ influences the relationship between stress and UTMA. Within the table, the moderation effect is illustrated by the interaction between stress and PetCO_2_ for both models. The SART1 model was statistically significant (R^2^ = 0.46, *p* = 0.05), confirming that PetCO_2_ moderated the relationship between subjective stress and UTMA. The overall moderation model for SART2 was not statistically significant (R^2^ = 0.27, *p* = 0.09) showing that PetCO_2_ did not moderate the relationship in this case.

The lower part of Table 3 shows the effect of stress on UTMA at different values of the moderator. The lowest value of PetCO_2_ given (31.30 mmHg) yielded a statistically significant result showing that the relationship between stress and UTMA is moderated at this level of PetCO_2_.

Table 4 presents the variances (R^2^ values) for the moderation models for SART1 and SART2 and the R^2^ values for the stress/PetCO_2_ interaction effect. During the SART1 test, 46% of the variance in UTMA was attributed to the stress experienced during the attention task (*p* = 0.01). Almost half (41%) of the variance was attributed to the interaction between stress and end-tidal CO_2_ (0.19/0.46). The variance level indicated in the overall SART2 model was not significant, but the stress/PetCO_2_ interaction was highly significant (*p* = 0.01).

Figure 1 is of a Johnson-Neyman plot showing the region of significance for the SART1 test (28.4 mmHg to 31.1 mmHg PetCO_2_). The significant PetCO_2_ values are within the hypocapnic range with most medical sources defining hypocapnia as less than 35 mmHg for partial CO_2_ pressure in arterial blood [25].

## 4. Discussion

The study firstly demonstrated that a sustained attention task can initiate a physiological stress response leading to increased UTMA and decreased PetCO_2_, (hyperventilation), which is consistent with previous research [6]. PetCO_2_ was also found to significantly moderate the relationship between attention-related stress and UTMA for SART1. In the context of this study, a moderation effect can be explained as a strengthening effect. This means that when a lower level of PetCO_2_ is reached during exhalation (and hyperventilation occurs), the stress experienced during the attention task significantly increases upper trapezius muscle activity. The overall moderation model was not significant for SART2, and PetCO_2_ did not, therefore, strengthen the relationship between attention-related stress and UTMA after the breathing protocol. The hypothesis, which proposed that a breathing technique can inhibit the moderation effect of PetCO_2_ thus, preventing an increase in UTMA due to a sustained attention task, can be partially accepted. The slowed breathing protocol prevented PetCO_2_ concentrations from decreasing (or hyperventilation occurring) in half the study group.

The breathing intervention seems to have had positive physiological effects on participants by weakening the interaction between stress and PetCO_2_. Levels of UTMA and breathing rate were lower after the breathing intervention for all participants (SART2), showing that the abdominal breathing was likely to have reduced hyperventilation and inhibition of the parasympathetic system within the study group, overall.

It is likely that those participants with decreased PetCO_2_ during SART2 did not carry out the breathing technique correctly. Studies have shown that novices learning breathing practices tend to exhale too forcefully causing a decline in PetCO_2_ [66]. Deliberate, slow-paced breathing can sometimes cause an increase in tidal volume that more than compensates for the decrease in respiration rate, thus, often producing hyperventilation [67]. Every individual has a “resonant frequency” breathing rate of about four to seven breaths per minute [68]. This level of coherent breathing has been shown to optimally balance the stress response for most adults [67]. In our experiment, breathing rate averaged at about 12 breaths per minute, which was considerably higher than the ideal rate. While this study did show promising effects of slowed breathing for some participants, a more comprehensive breathing training programme would need to be undertaken to ensure that all participants correctly modify their breathing technique and achieve an optimal level of coherent breathing.

Some researchers have hypothesised that PetCO_2_ mediates the relationship between stress and muscle activity [22], but this study suggests that PetCO_2_ is a moderator rather than a mediator, at least in this relationship. While a mediator is a variable the needs to be present for the relationship to occur, a moderator weakens, or as in this study, strengthens a relationship.

This finding that hyperventilation might be a moderator has broad and important implications for occupational health in industry. This means that improving breathing techniques in a working population may counteract the effects of stressful work such as sustained attention tasks, thereby reducing the level of muscle activity, a risk factor in the development of musculoskeletal disorders.

### 4.1. Implications for Industry

The findings of this study, along with previous studies, have found that monitoring work is stressful for operators. This does not align with the widely held view in industry that monitoring automated processes is not mentally demanding. An awareness of the physiological stress responses of monitoring work is necessary for industrial safety practitioners. Based on the results of this study, sustained attention work could be viewed as a potential psychosocial stressor which may be a contributor to the development of WRMSDs.

### 4.2. Limitations

As an observational study was undertaken as opposed to an experimental study, a control group was not employed. A control group would have helped to strengthen the finding that the breathing intervention resulted in lowered UTMA, breathing rate and hyperventilation for some study participants. The use of a comparison group would have clarified if other factors, e.g., practice effects on the SART task, or the time lapse of 30 min had any effect on the SART2 group results. Based on empirical studies relating to the effect of abdominal breathing techniques [35,40,62], however, it seems unlikely that the physiological differences after the breathing intervention can be solely attributed to other influences such as practice effects.

A further limitation is that some resource theorists have recently questioned whether the SART test is a better measure of impulse response than sustained attention [57]. While this may be the case, the SART test has been used across many disciplines as a sustained attention test over the past 30 years and proponents of the mindlessness theory of vigilance still advocate the use of the SART as a sustained attention test. SART has been frequently used in recent studies assessing the brain areas associated with attention failure [69,70,71,72]. The main outcome of these studies is that the brain areas active to an attentional failure in SART reflect brain activity during attention failures in everyday life. If this task has been shown to be an accurate measure of attentional failures, then it is difficult to say that it has no validity as an attention task. Along with this, the monitoring work carried out in industry is very diverse and it is likely that there are similar tasks to SART being undertaken.

### 4.3. Study Strengths

This study is unique in its use of an abdominal breathing intervention to reduce the physiological stress response resulting from a sustained attention task.

It also corroborates the existing hyperventilation theory by showing that hyperventilation plays a role in the relationship between psychosocial stress and muscle activity.

The study suggests that hyperventilation plays a moderating or strengthening role rather than a mediating role as previously proposed.

### 4.4. Opportunities for Future Research

It would be useful to perform industrial based studies measuring respiration and hyperventilation in addition to muscle activity during vigilance tasks. This would help to determine if hyperventilation is a moderator of the relationship between attention-related stress and increased muscular activity in workplace settings.

Sustained attention or supervisory monitoring work forms a dominant part of the role of operators in modern manufacturing, and as a psychosocial stressor, is a likely contributor to health problems, such as work related musculoskeletal disorders. It should, therefore, be included in workplace risk assessments.

As hyperventilation has been identified as a stress response that enhances the relationship between stress and muscle activity, targeting hyperventilation in the workplace may be a viable control measure in reducing the incidence of WRMSDs. The provision of stress management programmes which include training in breathing techniques might help reduce chronic hyperventilation in the workplace.

## 5. Conclusions

A sustained attention task resulted in a physiological stress response including hyperventilation and increased UTMA, which corroborates the findings of attentional resource theorists who advocate that monitoring work is stressful. This study highlights the role of hyperventilation as a moderator, which strengthens the relationship between stress and WRMSDs. The use of a simple abdominal breathing technique to weaken the effect of hyperventilation on this relationship was shown.

Key points:

- It is evident from this study that sustained attention work does initiate a physiological stress response including increased muscular activity and is therefore likely to be a risk factor in the development of WRMSDs.
- The study also provided evidence for hyperventilation being a moderator of the relationship between stress and muscle activity, rather than a mediator of the relationship, as previously proposed in the literature.
- A simple breathing intervention was partially successful in preventing hyperventilation, thereby weakening the relationship between stress and muscle activity.
- Attempts to reduce chronic hyperventilation in a workplace should help to alleviate the effects of sustained attention and other psychosocial stressors on musculoskeletal health.

## Figures and Tables

**Table 1:** Descriptive statistics of study variables.

Variable			C.I. of Mean					
	Condition	Mean (SD)	L.	U.	Homogeneity ^2^	Test Statistic	df	Sig.
UTMA (μV)	Baseline	12.24 (5.30)	10.00	14.47				
N = 24	SART1	22.46 (16.97)	15.29	29.63				
	SART2	15.17 (9.52)	11.15	19.18	0.03	χ^2^ = 7.07 ^1^	2	0.03 *
Breathing Rate (bpm)	Baseline	15.18 (3.34)	13.77	16.59				
N = 24	SART1	17.61(2.61)	16.51	18.72				
	SART2	15.60 (3.84)	13.98	17.22	0.37	F = 0.03	2	0.03 *
PetCO_2_ (mmHg)	Baseline	36.67 (3.64)	35.12	38.20				
N = 24	SART1	34.38 (3.09)	33.07	35.68				
	SART2	33.89 (4.53)	31.98	35.81	0.20	F = 0.03	2	0.03 *
Stress (SSSQ score)	Baseline	NA						
N = 24	SART1	0.08 (0.54)	−0.15	0.31				
	SART2	0.04 (0.87)	−0.33	0.40		T = 0.22	23	0.83

^1^ The upper trapezius muscle activity (UTMA) data were non-normally distributed so they were tested using the non-parametric Kruskal–Wallis test. ^2^ Homogeneity was tested using Levenes statistic. Breathing rate was measured in breaths per minute (bpm). PetCO_2_ is measured using the millimetres of mercury pressure measure (mmHg). The combined distress and worry subscales determined the SSSQ score for each participant. * Significant at the 0.05% level.

**Table 2:** SART2 test results—mean and standard deviation for each variable.

Variable	SART1	SART2 Group A (N = 12) with Increased PetCO_2_	SART2 Group B (N = 12) with Decreased PetCO_2_
BR	17.61 (2.61)	15.50 (1.38)	15.71 (2.83)
EMG	22.46 (16.97)	15.40 (10.72)	15.05 (8.62)
PetCO_2_	34.38 (3.09)	36.71 (2.55)	30.74 (4.36)
Stress	0.08 (0.54)	−0.21 (0.80)	0.28 (0.90)

BR = Breathing rate.

**Table 3:** Moderation model results for SART1 and SART2.

	SART1 (N = 24)							SART2 (N = 24)					
	Effect (B)	SEB	T	C.I. L.	U.	*p*		Effect (B)	SEB	t	C.I. L.	U.	*p*
Constant	96.60	32.39	2.98	29.02	164.16	0.05 *		−3.88	11.00	−0.23	−39.33	31.57	0.82
PetCO_2_	−2.20	0.94	−2.34	−4.17	−0.25	0.03 *		0.49	0.49	1.00	−0.53	1.51	0.33
Stress	−132.13	47.05	−2.81	−230.29	−33.96	0.01 **		45.65	17.33	2.63	9.50	81.80	0.02 *
PetCO_2_ X Stress	3.85	1.41	2.71	−0.89	6.80	0.01 **		−1.33	0.50	−2.69	−2.37	−0.29	0.01 **
Conditional effects of stress on UTMA at values of PetCO_2_													
PetCO_2_ (mmHg) SART1	Effect (B)	SEB	T	C.I. L.	U.	*p*	PetCO_2_ (mmHg) SART2	Effect (B)	SEB	t	C.I. L.	U.	*p*
31.30	7.35	5.46	1.34	−23.97	0.50	0.05 *	29.09	6.83	3.66	1.87	−0.80	14.47	0.08
34.38	1.63	4.44	0.37	−11.75	12.04	0.98	33.73	0.64	2.48	0.26	−4.53	5.81	0.79
37.47	−4.08	6.37	−0.64	−5.29	29.35	0.16	38.36	−5.54	3.08	−1.80	−11.97	0.88	0.08

C.I.: Confidence Interval,* Significant at the 5% level, ** significant at the 1% level.

**Table 4:** Moderation models R^2^ and interaction R^2^ for SART1 and SART2.

	Model R^2^	F-Statistic	*p*-Value	Interaction (Stress and PetCO_2_ R^2^)	F-Statistic	*p*-Value
SART1 Model (N = 24)	0.46	F(3,20) = 5.73	0.01	0.19	F(1,20) = 7.34	0.01 **
SART2 Model (N = 24)	0.27	F(3,20) = 2.43	0.09	0.26	F(1,20) = 7.24	0.01 **

** Significant at the 1% level.
